# Supplementary material for: Factor analysis of ancient population genomic samples
Source: Nat Commun. 2020 Sep 16;11:4661. doi: 10.1038/s41467-020-18335-6 (PMC7494920; doi:10.1038/s41467-020-18335-6)
Supplement: Supplementary file 1 — Supplementary Information [file 41467_2020_18335_MOESM1_ESM.pdf]

Supplementary Information for "Factor Analysis of Ancient Population Genomic Samples"

Olivier François[1] Flora Jay[2]

Authors' affiliations

[1] Université Grenoble-Alpes, Centre National de la Recherche Scientifique, Grenoble INP, Laboratoire TIMC-IMAG UMR 5525, 38000 Grenoble, France.

[2] Université Paris Sud, Centre National de la Recherche Scientifique, Laboratoire de Recherche en Informatique, Bâtiment 650 Ada Lovelace, 91405 Orsay Cedex, France.

Corresponding authors

flora.jay@lri.fr
olivier.francois@univ-grenoble-alpes.fr

Supplementary Note 1. Simplified R code for temporal factor analysis

```
temporal_fa = function(sample_ages, Y, k = 2, lambda = 1e-3){
 # sample_ages: Ages of samples (year BP/BCE or generations)
 # Y: Matrix of fully imputed/corrected genotypes
 # k: Number of factors
 # lambda: Hyper-parameter (usual range: 1 to 1e-6)

 # conversion of ages as elapsed times between 0 and 1
    Y <- t(scale(t(Y), center = TRUE, scale = FALSE))
    var_Y <- apply(Y, 1, FUN = var)
    range_ages <- max(sample_ages) - min(sample_ages)
    t_n <- 1 - (sample_ages - min(sample_ages))/range_ages
    t_n <- min(var_Y) +  (max(var_Y) - min(var_Y)) * t_n

 # Brownian covariance model
    n <- length(t_n)
    C <- matrix(NA, n, n)
    for (i in 1:n){
        for (j in 1:n)
            C[i,j] <- min(t_n[i], t_n[j])}

 # Compute eigenvectors and eigenvalues of C
    ec <- eigen(C)
    P_n <- ec$vector
    lambda_n <- ec$values

 # Estimate the latent matrix W
    D <- diag(sqrt(lambda/(lambda_n + lambda)))
    D_inv <- diag(sqrt((lambda_n + lambda)/lambda))
    sv <- svd(D %*% t(P_n) %*% Y, nu = k)
    U_n <- P_n %*% D_inv %*% sv$u %*% diag(sv$d[1:k])
    W_n <- U_n %*% t(sv$v[,1:k])

 # Return the latent matrix W and non-orthogonal factors
    return(list(w = W_n, u = U_n))
}
```

Supplementary Table 1. Proportion of variance explained by the first eigenvectors of the covariance model in two-way admixture estimates using Anatolian and Pontic steppe populations as sources.

	**Proportion of variance explained by distortions**	**Standard errors**
Germany LBK (N)	10.6%	10%
Austria LBK (N)	9.5%	9.9%
Greece Peloponnese	39.9%	22.6%
England EBA	20.9%	19.6%
Czech Rep. EBA	17.1%	16.7%
Germany EBA	13.4%	14%
Germany CW	7.6%	8.5%
England BA	20%	19.6%
Czech Rep. BA	22.1%	20.7%
Germany BA	15.6%	15.7%
Netherlands BA	18.2%	17.9%
Sweden Vikings	17.2%	17.2%
England Saxon	19%	17.3%
Hungary Langobard	17.7%	17.3%
Italy Langobard	17.4%	17.6%

Proportion of variance: Mean values over all polymorphic variants.
Standard errors: S.D. over all polymorphic variants.

Supplementary Table 2. Estimates of Yamnaya ancestry in two-way admixture models for European populations. FA scores and admixture coefficients using randomized dates (SD = 100y, 100 replicates) compared to reported dates.

	Admixture sd.	Bias toward 50%	Sq. correlation
Germany LBK (N)	0.0%	-0.1%	99.9%
Austria LBK (N)	0.0%	0.0%	99.9%
Greece Peloponnese	0.0%	0.0%	99.9%
England EBA	1.3%	1.8%	99.9%
Czech Rep. EBA	0.9%	1.3%	99.9%
Germany EBA	0.5%	0.7%	99.9%
Germany CW	0.1%	0.1%	99.9%
England BA	1.5%	1.5%	99.9%
Czech Rep. BA	1.2%	1.8%	99.9%
Germany BA	0.9%	2.6%	99.8%
Netherlands BA	1.3%	1.5%	99.9%
Sweden Vikings	0.2%	0.7%	99.9%
England Saxon	0.2%	0.0%	99.9%
Hungary Langobard	0.2%	0.5%	99.9%
Italy Langobard	0.1%	-0.2%	99.9%

Admixture sd.: standard deviation of population average admixture coefficients over 100 runs with uncertain ages.
Bias toward 50%: Observed bias compared to estimates obtained as average of 95.4% date range in calBP (defined as 1950 CE). Positive values were biased toward 50%. Negative values were biased toward 0% or 100%.
Sq. correlation: Averaged squared correlation between factor 1 scores with and without age uncertainty.

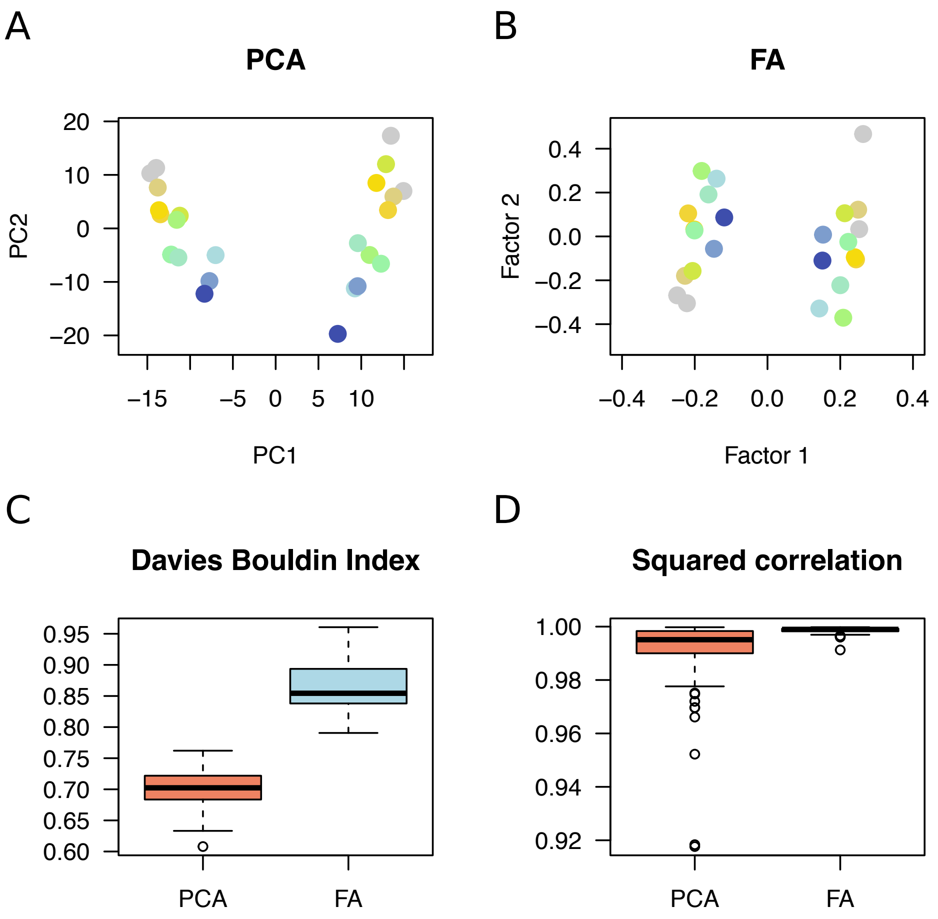

Supplementary Figure 1. Simulation of two-population models. Twenty-four samples with ages ranging from 0 (present, grey color) to 1,000 generations (past, dark blue color) were simulated. A) Typical PC plot for observed samples (populations are separated according to their sign on PC1), B) Factor analysis plot showing correction for temporal drift (populations are separated according to their sign on Factor 1), C) Coalescent simulations: Davies-Boulding clustering index for PCA and FA results ($n = 100$ simulated data sets, higher values indicate better clustering), D) Generative model simulations: Squared correlation between PC1/Factor 1 and a simulated (true) factor having two modes ($n = 100$ simulated data sets). Boxplot parameters: minimum, median, interquartile range, $1.5 \times$ 75th percentile or maximum.

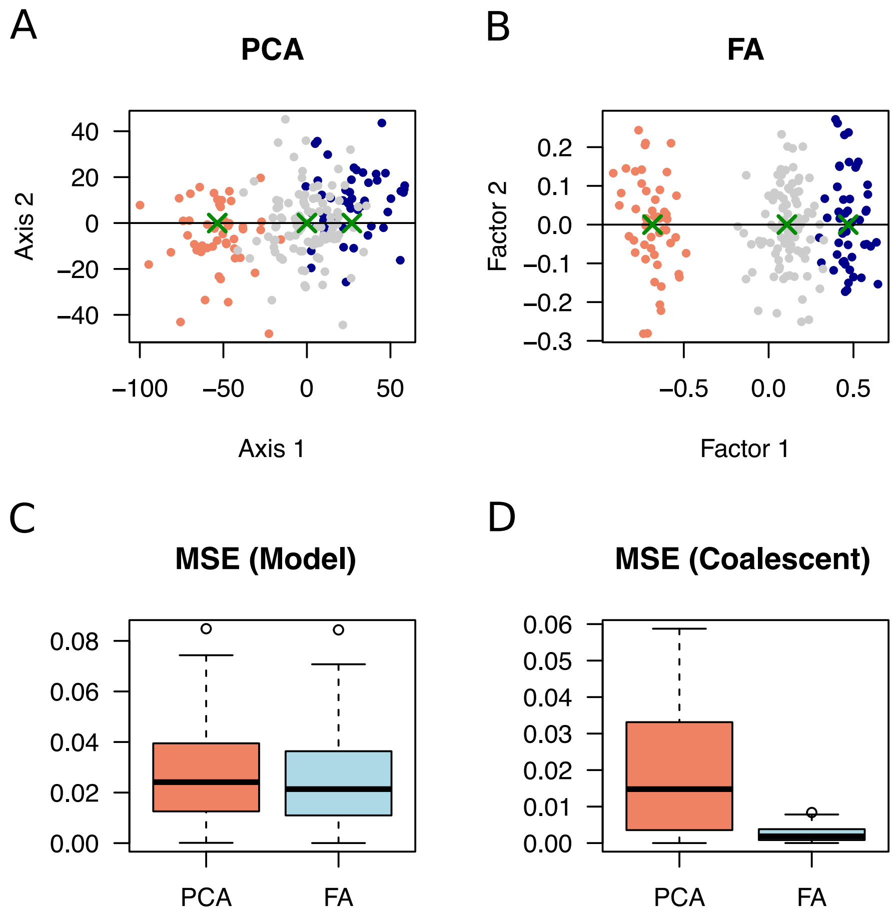

Supplementary Figure 2. Admixture model simulation showing shrinkage in PC projections. Simulation of two-population admixture models with 25-75 % admixture proportions. Two hundred samples with ages equal to 0 (present-day admixed individuals, grey color) and 1,000 generations (ancestors, orange and blue colors) were simulated. A) Plot of projections of ancient samples onto the present-day admixed population exhibiting a shrinkage effect, B) Factor analysis plot showing correction for shrinkage and recovery of simulated proportions, C) Generative model simulations: Mean square error (MSE) for estimates of admixture proportions from PC projections and FA plots ($n = 100$ replicates), D) Coalescent simulations: Mean square error (MSE) for estimates of admixture proportions from PC projections and FA plots ($n = 100$ replicates). Green crosses represent population centers from which admixture estimates were computed. Boxplot parameters: minimum, median, interquartile range, 1.5 $\times$ 75th percentile or maximum.

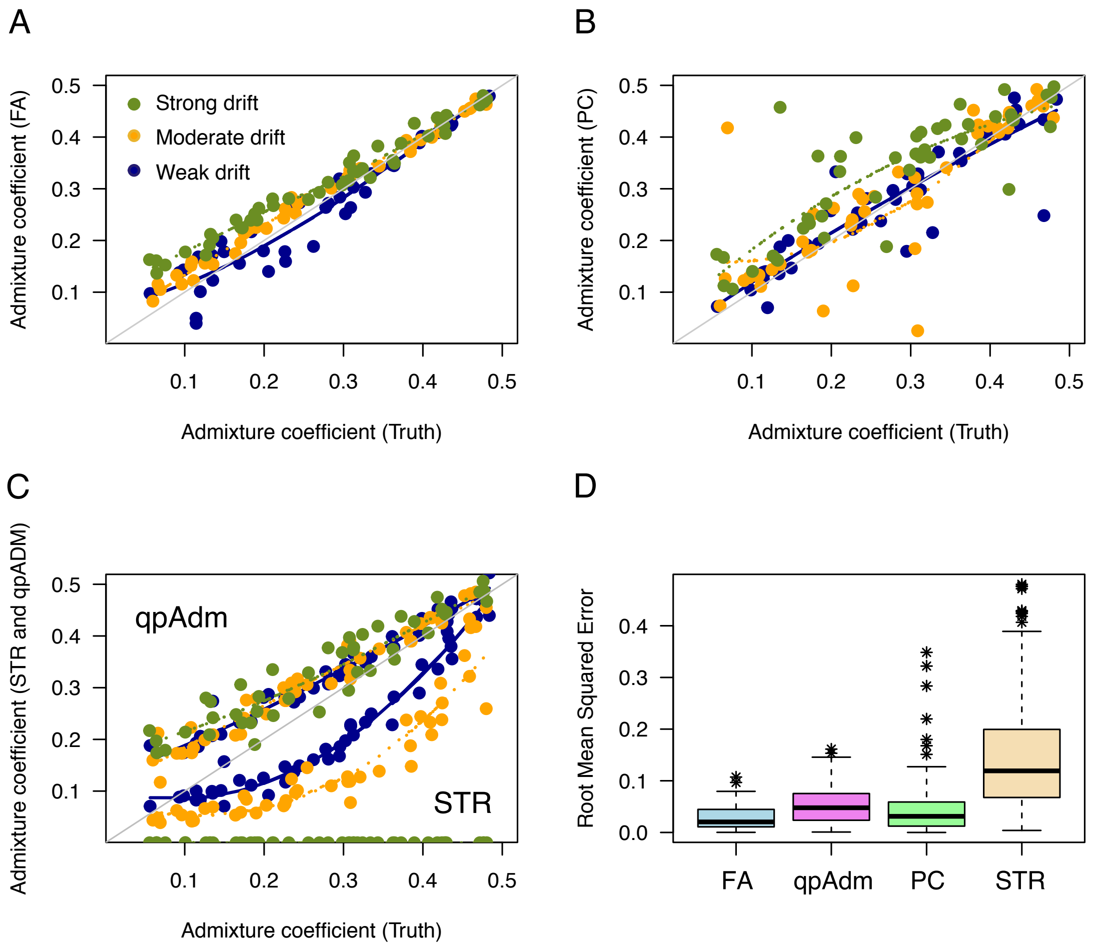

Supplementary Figure 3. FA ancestry estimates compared to other approaches (lower divergence value). Inference of ancestry proportions in two-population admixture models. Two hundred samples including 100 present-day individuals and 100 ancestors were simulated. The level of divergence between ancestral populations was equal to $F_{ST} = 0.05$. A) Ancestry estimates from FA, B) Ancestry estimates from PC projections, C) Ancestry estimates from qpAdm (above diagonal) and STRUCTURE (below diagonal), D) Root mean squared errors for ancestry estimates from four methods. FA: Factor Analysis, qpAdm: see text, PC: Projections on PCs of admixed samples, STR: STRUCTURE with sparse non-negative matrix factorization. Boxplots computed on $m = 135$ replicates (minimum, median, interquartile range, 1.5 × 75th percentile or maximum, 540 analyses).

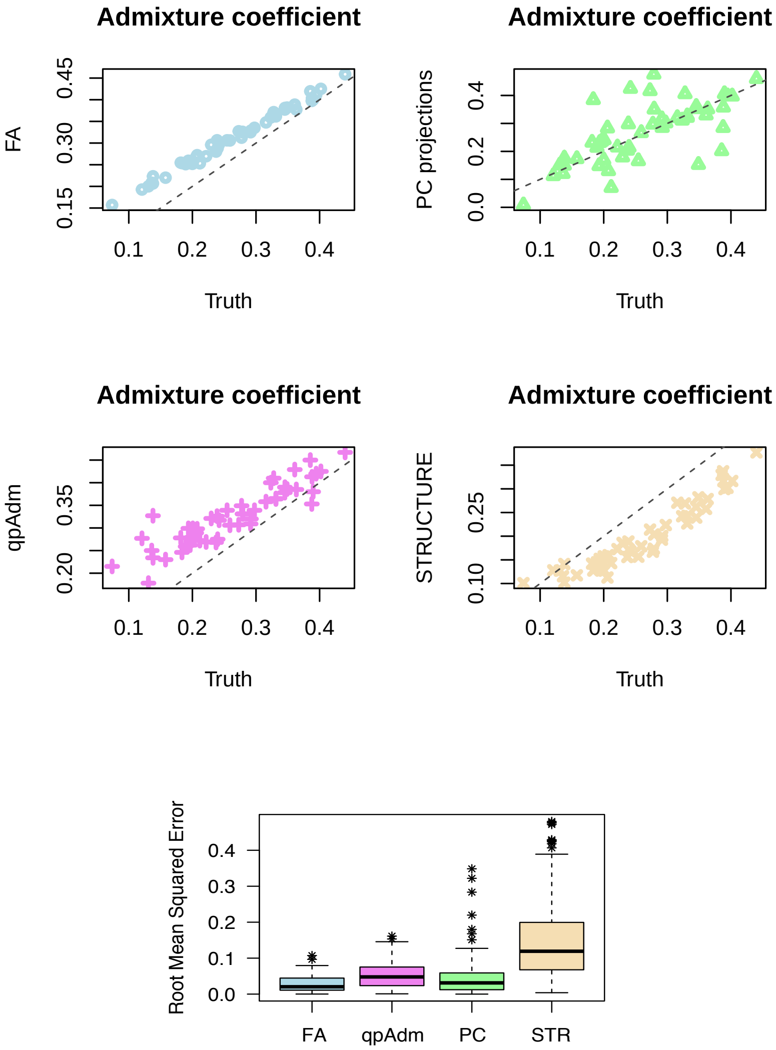

Supplementary Figure 4. Inference of ancestry proportions in two-population admixture models with realistic simulation parameters. Comparisons of ancestry estimates from factor analysis (FA), qpAdm, projections on PCs of admixed samples, and STRUCTURE (STR)implemented with sparse NMF. The level of divergence between ancestral populations was equal to $F_{\text{ST}} = 0.05$, the time since admixture was around 0.25 of the oldest sample date, and the amount of drift was low ($\lambda = 0.15$). These values were related to the admixture of European Bronze Age populations, considering a date of admixture around 5ky BP, an uncorrected F_{ST} between Yamnaya and Anatolian samples as observed in the data, and an upper bound on noise-to-drift ratios. Truth: Ground truth estimate computed from the simulation, RMSE: Root mean squared error. Boxplots computed on $m = 45$ replicates (minimum, median, interquartile range, 1.5 $\times$ 75th percentile or maximum).

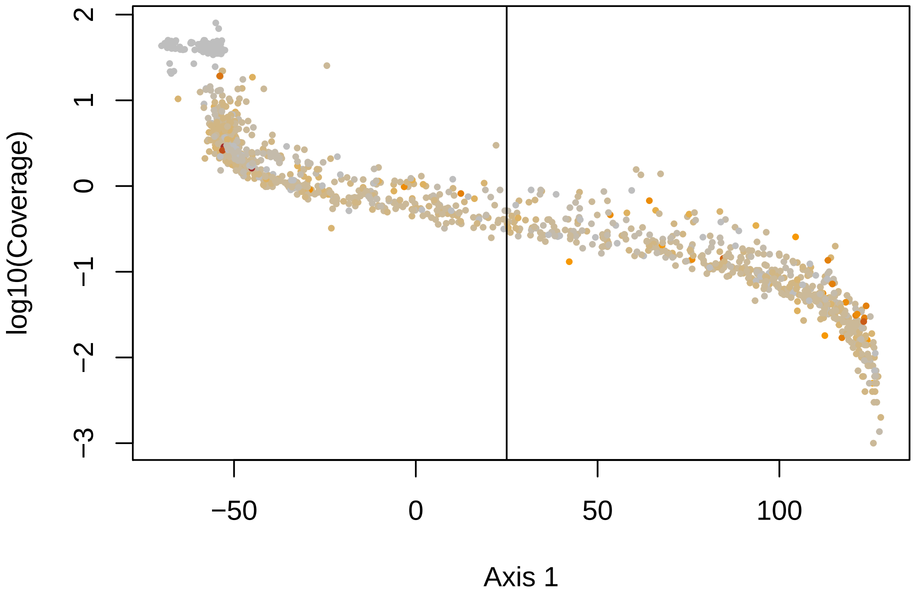

Supplementary Figure 5. Effect of coverage on a naive PC analysis of ancient and present-day samples. First axis of a principal component analysis of Eurasian genomes from a merged data set of ancient samples and European genomes from The 1,000 Genomes and Simons Genome Diversity data sets. The y-axis represents the genomic coverage of samples. Colors represent sample ages ranging between 0 and 35,000 years cal BP and from grey and yellow to red. The vertical line indicates the threshold below which samples were included in our analysis. Present-day individuals had the highest coverage. Coverage was weakly correlated to sample age otherwise.

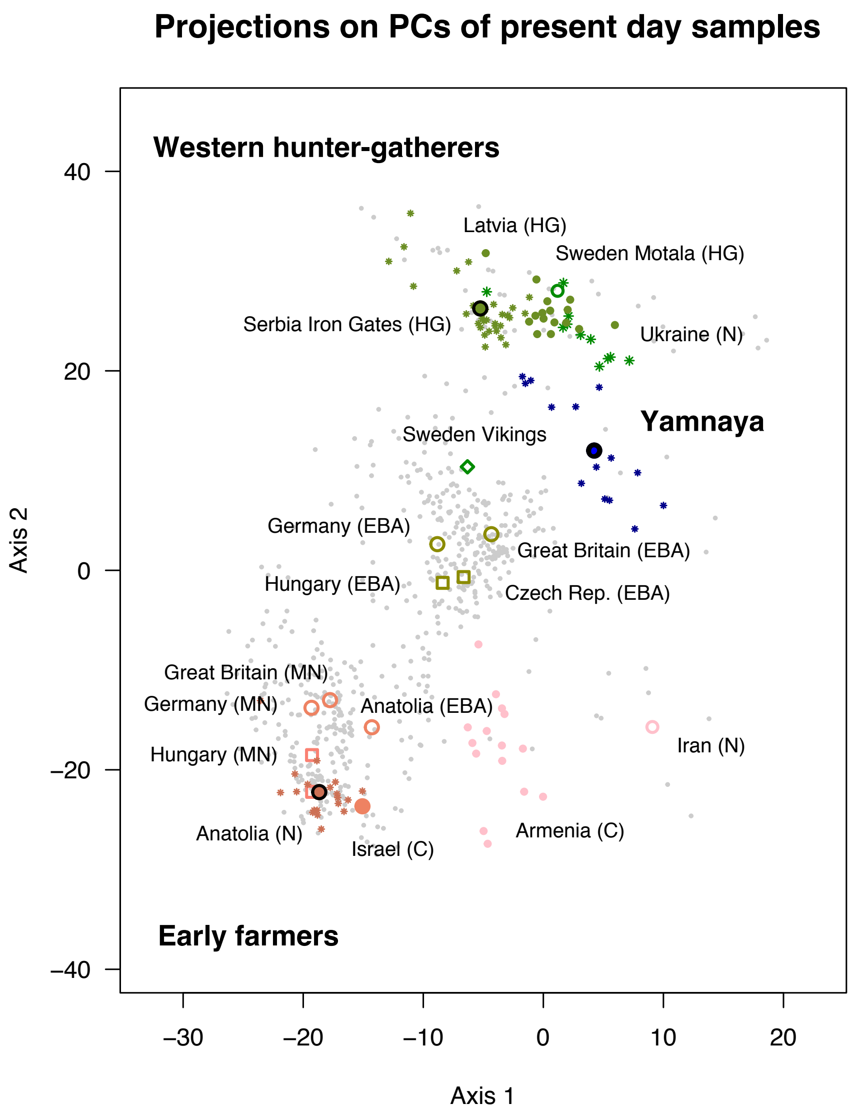

Supplementary Figure 6. Projections of ancient genomes on principal components of present-day samples. Projections of 704 ancient genomes with ages more recent than 12 ky cal BP on principal components of 521 present-day European genomes. HG: Hunter-Gatherers, N: Neolithic, MN: Middle Neolithic, C: Copper Age, EBA: Early Bronze Age. Ancient samples are represent by grey or colored points. Larger points represent population centers. Present-day samples are not represented.

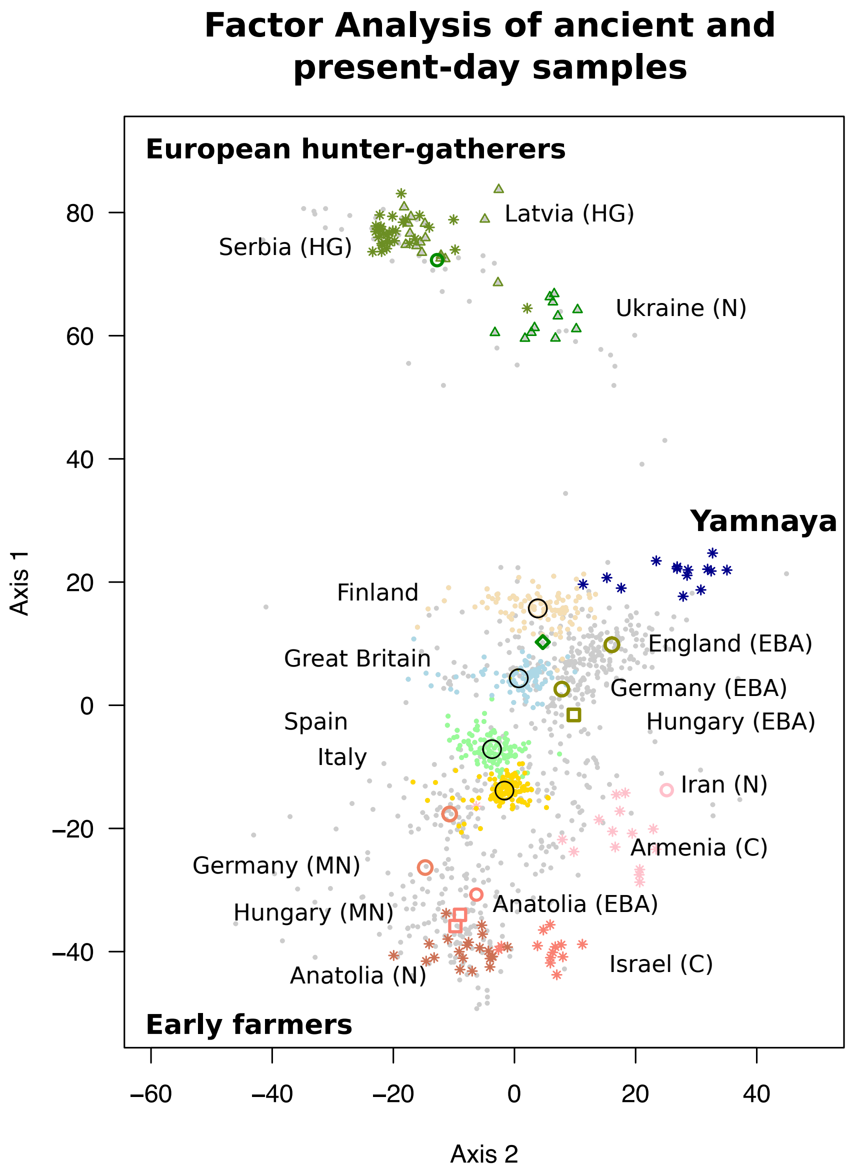

Supplementary Figure 7. Factor analysis of ancient and present-day samples. Factor analysis of 704 ancient genomes and 521 genomes from the 1k Genomes Project data set (ages more recent than 14 ky cal BP). The squared-correlation between Axis 1 and the first principal component of a PCA on the present-day samples was equal to $\rho^2 = 0.91$, ($P < 10^{-16}$, one-sided F-test with 1 and 519 df). HG: Hunter-Gatherers, N: Neolithic, MN: Middle Neolithic, C: Copper Age, EBA: Early Bronze Age. Large black circle: present-day samples.

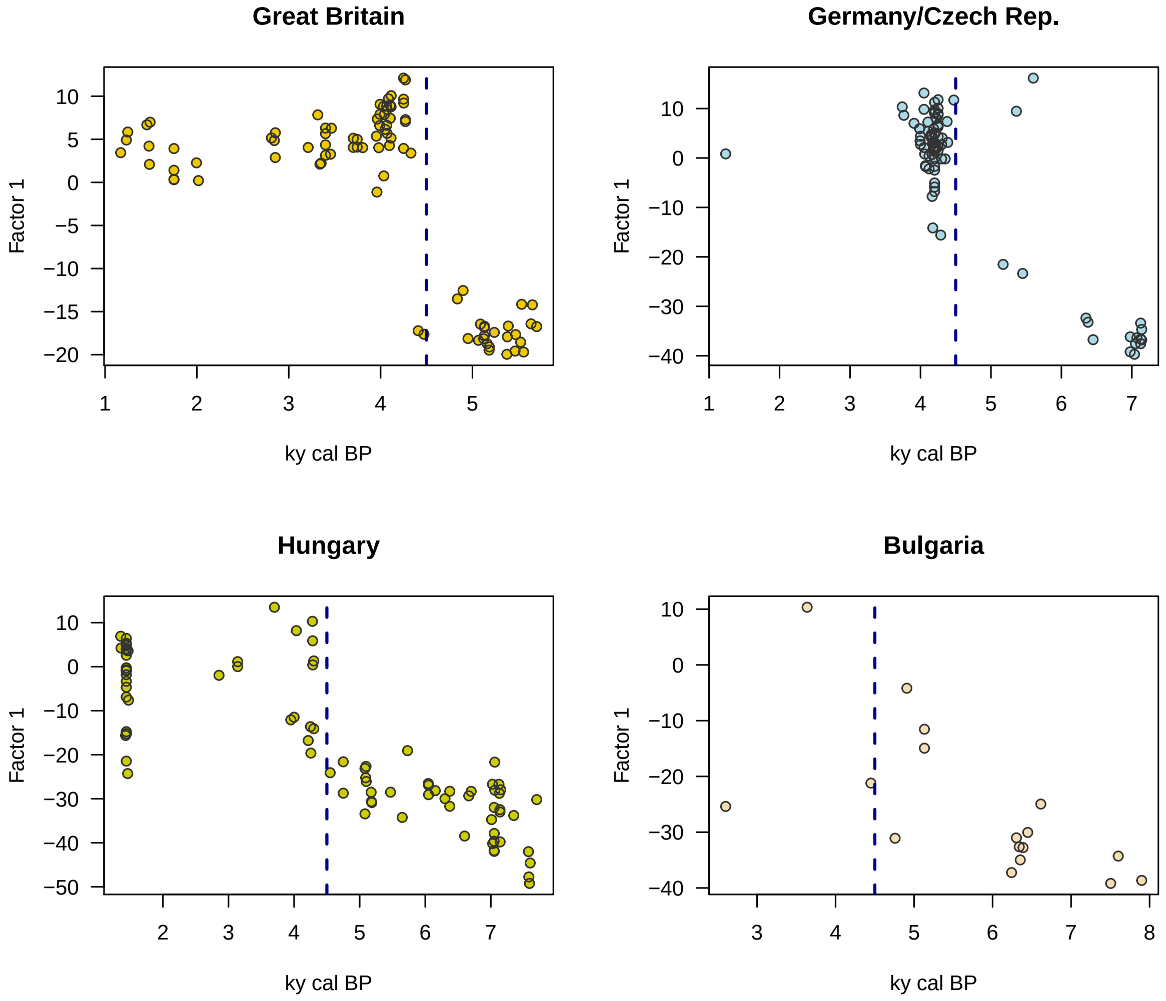

Supplementary Figure 8. Factor 1 as a function of age for samples from four geographic regions. Factor 1 in FA represents the relative genetic contribution of ancient Anatolian farmers and hunter-gatherers to ancient European samples. The FA supports a resurgence of hunter-gatherer ancestry in late neolithic samples from Central Europe (dashed line: 4,500 years BP).

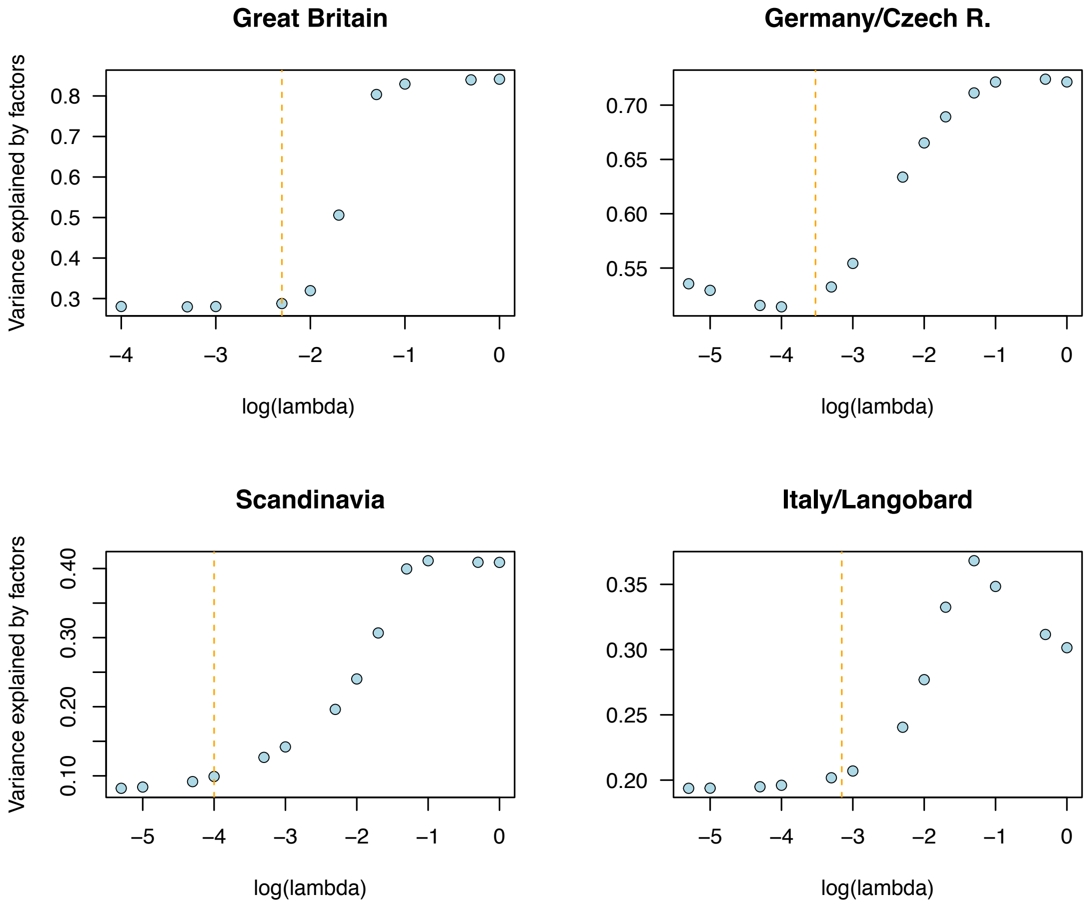

Supplementary Figure 9. Variance of sample ages explained by the first latent factors and choice of the drift parameter in FA. Factor analysis of samples from four geographic regions, considering Anatolian Neolithic and Pontic steppe samples as sources in two-way admixture analyses. In those analyses, regression analyses were performed with sample age considered as a response variable explained by latent factors 1 and 2. The drift parameter (λ) was selected as the largest value for which the variance explained by factor 2 was removed.

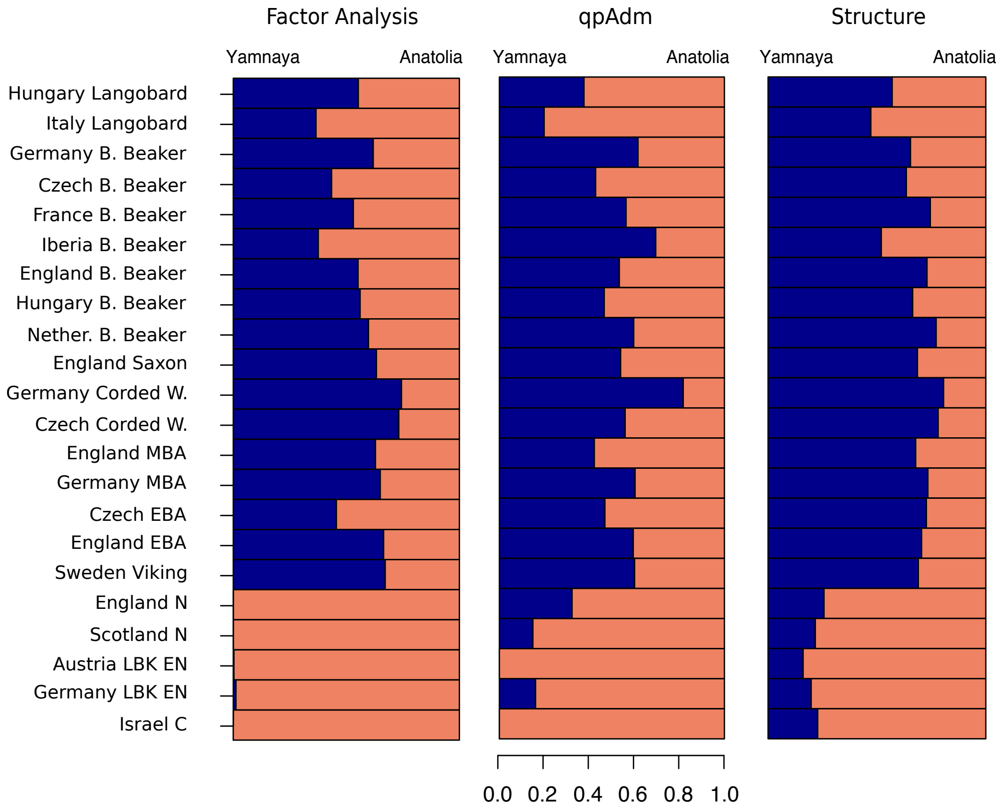

Supplementary Figure 10. Two-way admixture: Steppe ancestry in ancient European samples. Steppe ancestry coefficients (dark blue color) estimated from FA using Yamnaya and Anatolia (N) as ancestral groups, qpAdm with Yamnaya and Anatolia (N) as ancestors, Yoruba, Russia Sidelkino (HG) and France-Ranchot (WHG) as outgroups, and STRUCTURE (sparse NMF) using Yamnaya and Anatolia (N) as ancestral groups. N: Neolithic, BA: Bronze Age, C: Chalcolithic, E: Early, M: Middle.

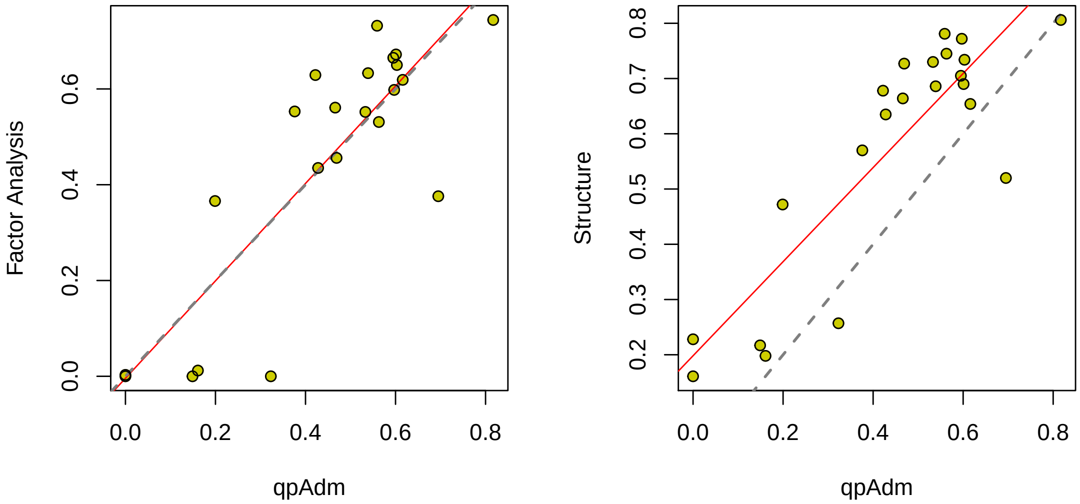

Supplementary Figure 11. FA ancestry estimates compared with F-statistics (qpAdm) and STRUCTURE in two-way admixture analyses. The proportions of steppe ancestry for ancient European samples from the Neolithic period to the Iron Age and the Early Middle-Ages were computed from FA, qpAdm and STRUCTURE (sparse NMF). Except for neolithic samples (with values equal to zero), FA estimates agreed with F-statistics. STRUCTURE overestimated the proportion of steppe ancestry. The program qpAdm was used with Yoruba, Russia Sidelkino (EHG), France-Ranchot (WHG) as outgroups. Dashed line: equal values (diagonal), red line: linear regression.

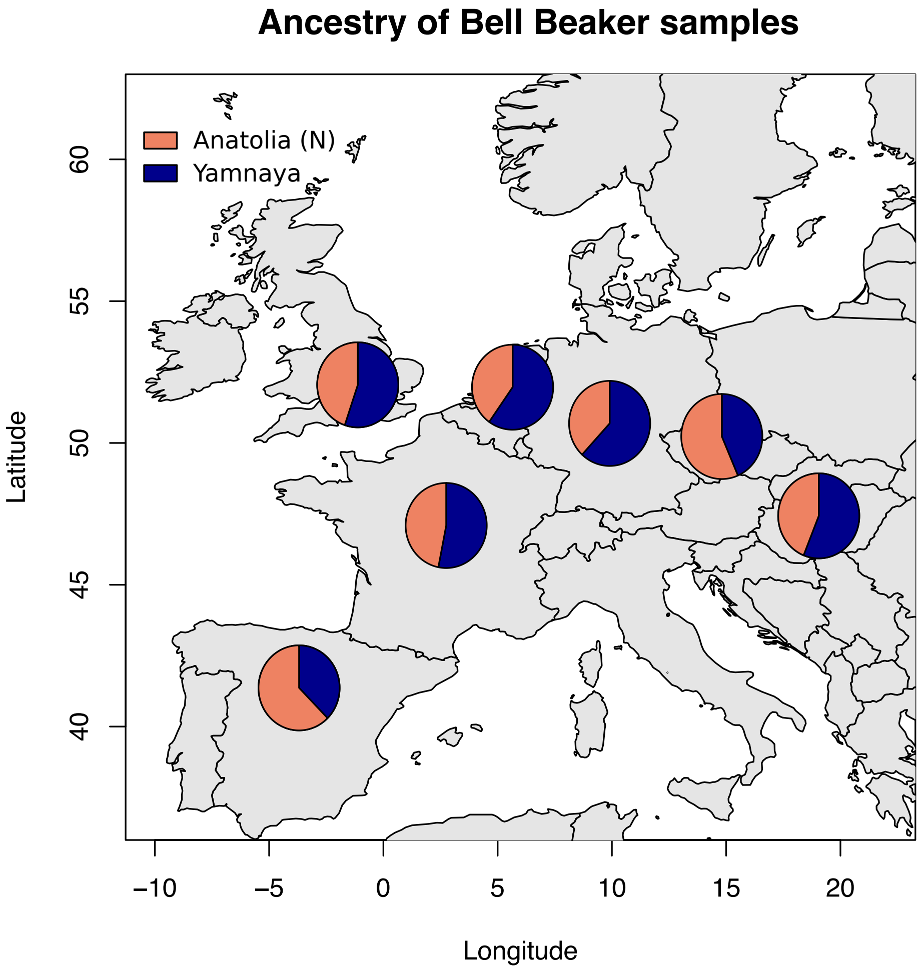

Supplementary Figure 12. Ancestry of Bell Beaker samples computed from FA (two-way admixture). Bronze Age Bell Beaker samples from Hungary (2), Czech Republic (22), Germany (26), Netherlands (8), France (3), Iberia (4) and England (16). The analyses includes samples from early farmers from Anatolia (21) and Yamnaya (14).

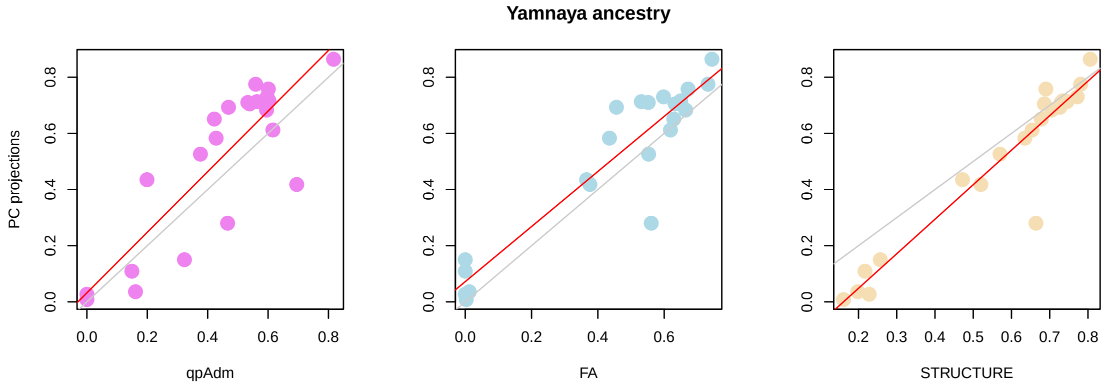

Supplementary Figure 13. Proportions of steppe ancestry obtained from PC projections (two-way admixture analyses). Two-way admixture estimates obtained from PC projections on present-day samples are compared with qpAdm, FA, and STRUCTURE (sparse NMF) estimates for 22 ancient samples dating from the Neolithic period to the Iron Age and the Early Middle-Ages. Ancestral populations: Yamnaya (Samara) and Anatolia (N). The ancestry estimates from PC projections over-estimated FA and qpAdm values, and better agreed with STRUCTURE estimates. The program qpAdm was used with Yoruba, Russia Sidelkino (EHG), France-Ranchot (WHG) as outgroups. Grey line: diagonal, red line: linear regression.

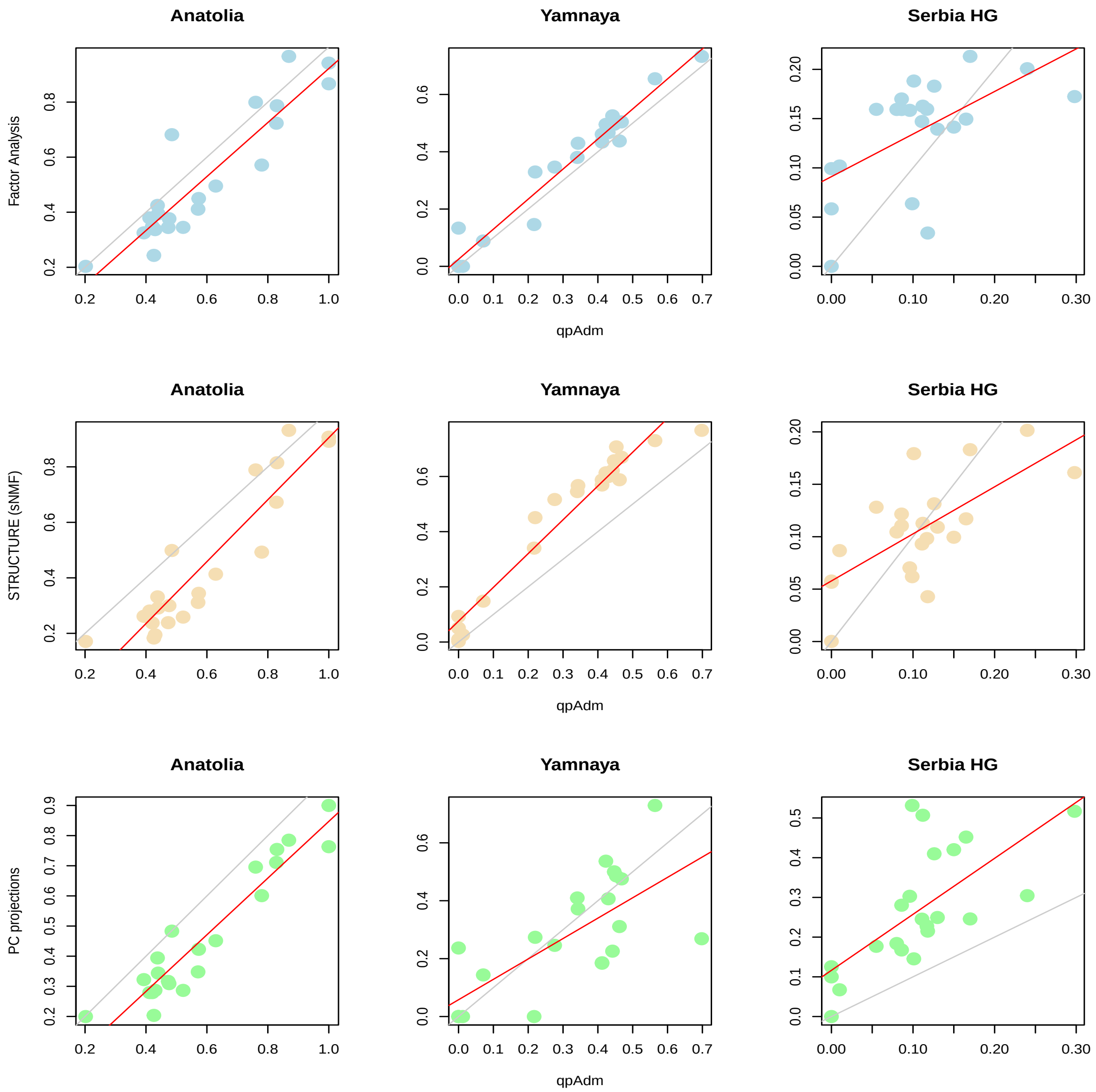

Supplementary Figure 14. Comparaison of ancestry estimates in three-way admixture analyses. Top row: FA estimates correlate with qpAdm estimates, providing slightly higher coefficients of Yamnaya ancestry. **Middle row**: STRUCTURE (sNMF) coefficients over-estimate Yamnaya ancestry. **Bottom row**: PC estimates exhibit over-dispersion of samples within the Yamnaya-WHG axis. FA was implemented with $\lambda = 0.05$, qpAdm was implemented with Yoruba, Russia Sidelkino (EHG), France-Ranchot (WHG), Aleut and Altaian samples as outgroup samples. Grey line: diagonal, red line: linear regression.

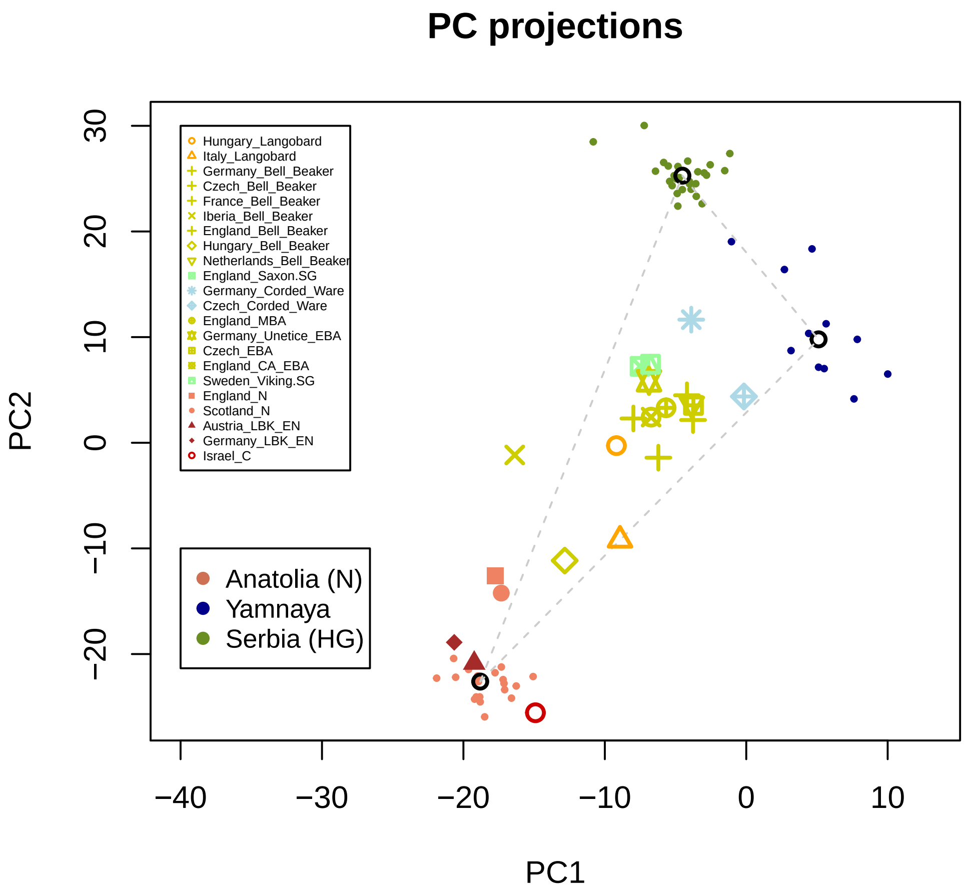

Supplementary Figure 15. PC projections of 22 ancient European samples. Only the centers of population samples are shown.

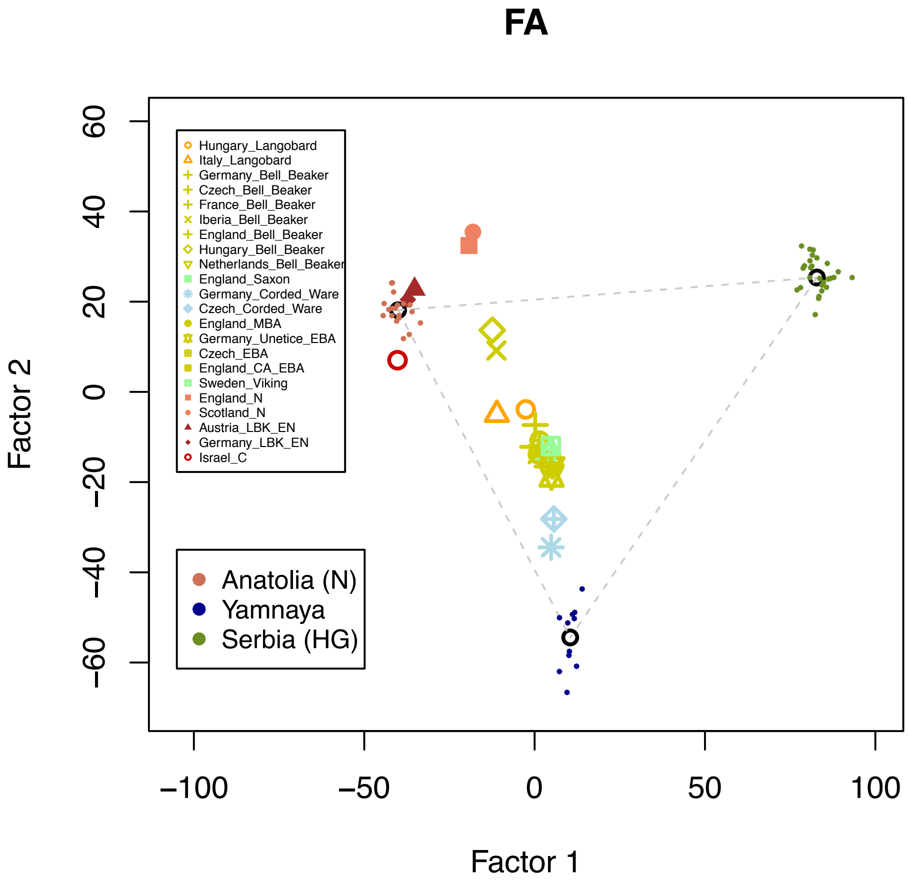

Supplementary Figure 16. FA analyses of 22 ancient European samples. Only the centers of population samples are shown. FA was implemented with $\lambda = 0.05$.
