# Supplementary material for: Factor analysis of ancient population genomic samples
Source: Nat Commun. 2020 Sep 16;11:4661. doi: 10.1038/s41467-020-18335-6 (PMC7494920; doi:10.1038/s41467-020-18335-6)
Supplement: Supplementary file 3 — Description of Additional Supplementary Files [file 41467_2020_18335_MOESM3_ESM.pdf]

Description of Additional Supplementary Files

Supplementary Data 1

Description: Sample information and metadata
