# Supplementary material for: Factor analysis of ancient population genomic samples
Source: Nat Commun. 2020 Sep 16;11:4661. doi: 10.1038/s41467-020-18335-6 (PMC7494920; doi:10.1038/s41467-020-18335-6)
Supplement: Supplementary file 5 — Reporting Summary [file 41467_2020_18335_MOESM5_ESM.pdf]

nature research

Corresponding author(s): Olivier François

Last updated by author(s): Jul 21, 2020

Reporting Summary

Nature Research wishes to improve the reproducibility of the work that we publish. This form provides structure for consistency and transparency in reporting. For further information on Nature Research policies, see our Editorial Policies and the Editorial Policy Checklist.

Statistics

For all statistical analyses, confirm that the following items are present in the figure legend, table legend, main text, or Methods section.

n/a	Confirmed	
☐	☒	The exact sample size (*n*) for each experimental group/condition, given as a discrete number and unit of measurement
☐	☒	A statement on whether measurements were taken from distinct samples or whether the same sample was measured repeatedly
☐	☒	The statistical test(s) used AND whether they are one- or two-sided<br>*Only common tests should be described solely by name; describe more complex techniques in the Methods section.*
☐	☒	A description of all covariates tested
☐	☒	A description of any assumptions or corrections, such as tests of normality and adjustment for multiple comparisons
☐	☒	A full description of the statistical parameters including central tendency (e.g. means) or other basic estimates (e.g. regression coefficient) AND variation (e.g. standard deviation) or associated estimates of uncertainty (e.g. confidence intervals)
☐	☒	For null hypothesis testing, the test statistic (e.g. *F*, *t*, *r*) with confidence intervals, effect sizes, degrees of freedom and *P* value noted<br>*Give P values as exact values whenever suitable.*
☐	☒	For Bayesian analysis, information on the choice of priors and Markov chain Monte Carlo settings
☒	☐	For hierarchical and complex designs, identification of the appropriate level for tests and full reporting of outcomes
☐	☒	Estimates of effect sizes (e.g. Cohen's *d*, Pearson's *r*), indicating how they were calculated

Our web collection on statistics for biologists contains articles on many of the points above.

Software and code

Policy information about availability of computer code

Data collection	*Provide a description of all commercial, open source and custom code used to collect the data in this study, specifying the version used OR state that no software was used.*
Data analysis	R 3.6.3 for OS X 10.11, R 3.6.4 for Linux, R package LEA 2.4 from Bioconductor, lfmm 1.0 (https://github.com/bcm-uga/lfmm), R package admixr 0.7.1, ADMIXTOOLS version 6.0 for macOSX and Linux, R package tfa: https://github.com/bcm-uga/tfa, msprime version 0.7.6 for Linux

For manuscripts utilizing custom algorithms or software that are central to the research but not yet described in published literature, software must be made available to editors and reviewers. We strongly encourage code deposition in a community repository (e.g. GitHub). See the Nature Research guidelines for submitting code & software for further information.

Data

Policy information about availability of data

All manuscripts must include a data availability statement. This statement should provide the following information, where applicable:

- Accession codes, unique identifiers, or web links for publicly available datasets
- A list of figures that have associated raw data
- A description of any restrictions on data availability

Data were extracted from a public data set consisting of genotypes for 5,081 ancient and present-day individuals compiled from published papers and available from David Reich's repository (February 2019, v.37.2): reich.hms.harvard.edu. All data have already been published, and have permissions appropriate for fully public data release. The IDs of samples included in our study were provided in Supplementary File 1, and the corresponding DOIs were referenced from the database.

Field-specific reporting

Please select the one below that is the best fit for your research. If you are not sure, read the appropriate sections before making your selection.

☐ Life sciences ☐ Behavioural & social sciences ☒ Ecological, evolutionary & environmental sciences

For a reference copy of the document with all sections, see nature.com/documents/nr-reporting-summary-flat.pdf

Ecological, evolutionary & environmental sciences study design

All studies must disclose on these points even when the disclosure is negative.

Study description	This study presented a new data analysis method for ancient DNA, used simulations to evaluate the method, and applied the method to ancient human DNA data taken from previously published studies.
Research sample	The ancient DNA data contained 143,081 SNPs genotyped for 521 present-day European individuals from the 1 Thousand Genomes and Simons projects, and for 704 ancient Eurasian samples taken from previous studies (human data). Data were extracted from a public data set consisting of genotypes for 5,081 ancient and present-day individuals compiled from published papers and available from David Reich's repository (February 2019, version 37.2): reich.hms.harvard.edu. All data have already been published, and have permissions appropriate for fully public data release. The IDs of samples included in our study were provided in Supplementary File 1, and the corresponding DOIs were referenced from the database. The samples included in the study were chosen on the basis of quality, higher genomic coverage, and to have ages more recent than 13,980 years. The samples were meant to be representative of ancestry analyses from ancient human DNA data, from the Neolithic to more recent periods.
Sampling strategy	We used all the publicly available samples at the time the study was started
Data collection	We did not collect the data.
Timing and spatial scale	The data were collected and described in several other studies published between 2010 and 2019 (public data). Geographic scale: Eurasia, Period studied: 14k years BP to present.
Data exclusions	Lower quality and coverage data were excluded from analyses.
Reproducibility	Reproducibility was guaranteed by the availability of computer codes replicating our analyses. All data were publicly available.
Randomization	We did not performed random allocation of samples across treatment groups.
Blinding	Blinding is not relevant to archaeological samples and to the genealogical analyses performed in this study.

Did the study involve field work? ☐ Yes ☒ No

Reporting for specific materials, systems and methods

We require information from authors about some types of materials, experimental systems and methods used in many studies. Here, indicate whether each material, system or method listed is relevant to your study. If you are not sure if a list item applies to your research, read the appropriate section before selecting a response.

Materials & experimental systems

n/a	Involved in the study
☒	☐ Antibodies
☒	☐ Eukaryotic cell lines
☒	☐ Palaeontology and archaeology
☒	☐ Animals and other organisms
☒	☐ Human research participants
☒	☐ Clinical data
☒	☐ Dual use research of concern

Methods

n/a	Involved in the study
☒	☐ ChIP-seq
☒	☐ Flow cytometry
☒	☐ MRI-based neuroimaging
